# Ensuring Effective Public Health Communication: Insights and Modeling Efforts From Theories of Behavioral Economics, Heuristics, and Behavioral Analysis for Decision Making Under Risk

**DOI:** 10.3389/fpsyg.2021.715159

**Published:** 2021-10-13

**Authors:** Darren J. Edwards

**Affiliations:** Department of Public Health, Policy, and Social Sciences, Swansea University, Swansea, United Kingdom

**Keywords:** prospect theory, dual process theory, relational frame theory (RFT), public health messaging, machine learning

## Abstract

Public health (PH) messaging can have an enormous impact on shaping how individuals within society behave, and can ensure it is in a safe and responsible way, consistent with up-to-date evidence-based PH guidelines. If done effectively, messaging can save lives and improve the health of those within society. However, unfortunately, those within Government PH bodies typically have little training about how to effectively represent PH messages in a way that is consistent with psychological theories of cognitive bias, in order to avoid cognitively biasing the public through their messages. As a result of this, inadequate representation of PH messages can result, which can often lead to cognitive bias in those from the public who read or listen to the message information. This can lead to poor decision making of the pubic as a whole, which can then further lead to harm and even death of public members as a result of these poor decisions. One way to minimize the problem of bias in decision making is to explore psychology theories that model how bias can occur from PH messaging, and identify ways in which PH agencies can utilize such approaches to improve the effectiveness of their messages. Previous focus has been largely on behavioral economic theories, however, here, other accounts are offered in addition to these. These include theories of heuristics and theories from the behavior analysis domain, which may increase the predictive power of modeling bias, and have applications for how best to represent PH message information which minimize bias.

## Introduction

Effective public health (PH) messaging can have a very positive impact on the health of those within society who follow the evidence-based guidelines contained within the messages (Chambers et al., 2005; Rimal and Lapinski, 2009; Bassil and Cole, 2010). Similarly, PH messaging that is ineffective can lead to higher stress and stigma (Puhl and Heuer, 2010; Coupet et al., 2021) as well as facilitate cognitive bias (Garcia-Retamero and Galesic, 2010; McKee and Stuckler, 2010; Luz et al., 2020).

Recent examples of important PH messages where there was confusion due to ineffective or inconsistent messaging include; whether there was a need to wear masks during the COVID-19 pandemic (Cheng et al., 2020; Kolstoe, 2020; Tang, 2020), effectively convincing and countering anti-vaccination messaging (Hobson-West, 2003; Burki, 2020; Ransing et al., 2021), as well as countering misinformation about links between 5G and COVID-19 (Kyriakidou et al., 2020). PH agencies around the world often employ fact checkers to counter misinformation (Morgenstern and Pizer, 2007; Hameleers and van der Meer, 2020). However, online misinformation is widespread and difficult to track and contain (Collier, 2018; Swire-Thompson and Lazer, 2020; Zucker, 2020), and so these counter efforts against misinformation usually achieve only a limited impact.

One of the reasons for why PH messages can be ineffective, is that PH agencies often have to simplify very complicated science into simple bite-sized messages that the public can understand quickly (Bosk et al., 2009; Ahn et al., 2011). However, this can inadvertently lead to oversimplification and incorrect public assumptions, which may lead to PH harm.

In addition to these problems, there may be more fundamental issues which relate to PH messaging and are currently overlooked by PH agencies. Specifically, this relates to how these messages can lead to cognitive bias, and under what context these biases occur. Some examples of possible biases which can occur when communicating information in a PH message include: (1) anchoring, whereby an individual relies too heavily on some initial piece of information they receive when making subsequent decisions, even when given new information which may conflict with the initial information (Zhang et al., 2007; Lieder et al., 2018); (2) automation bias, the tendency to over rely on, and have confidence in, automated processes, such as programs that filter information or provide diagnostics, to make decisions. These of course can be useful, but they can also introduce biases if over relied on, and especially in cases where the processes of automation are not supervised (Goddard et al., 2011); (3) the gambler’s fallacy (Hsee and Zhang, 2004; Barron and Leider, 2010; Lieder et al., 2018) where the individual believes that a certain random event is more or less likely to occur because of a previous event which occurs in a similar setting. For example, someone may believe that in a coin toss, the coin will land on heads if the coin had landed on tails in a previous coin toss, when the real odds are still an even 50% chance despite previous outcomes; and (4) the conjunction fallacy (Tentori et al., 2004; Franco, 2009), a bias whereby an individual believes that certain events will have an increased likelihood of co-occurring than occurring alone (such as hailstones with a thunderstorm) when in reality, the probability of such things co-occurring can only be the same or lower than of occurring alone.

There are of course many more types of bias, and much of the reason for why cognitive biases occur have been thought to relate to the limited resources of the cognitive system such as short term memory, attention, and information processing capacity (Franconeri et al., 2013; Korteling et al., 2018). From this perspective, Neisser (1976) defined cognition as a dynamic information processing system which is designed to transform, reduce, elaborate, store, recover, and use sensory input. This perspective ontologically explores cognition through information theory (Shannon, 1948), which defines the smallest quantifiable unit of information as one bit of data. In this case, one decision between two equally likely outcomes (e.g., choosing heads or tails in a coin flip), can be thought of as one bit of information.

Biases, from this perspective, are thought to be due to the limited number to bits of information an individual can store and process at one time, so require cognitive shortcuts, called heuristics to process this information (Tobin, 2009; MacLean and Dror, 2016). For example, a study (Chaiken, 1980) found that the recipient was more likely to be persuaded by a message through a simple heuristic rule when not utilizing all of their information processing resources, and the message content when the recipient could allocate more information processing resources to the message. In another example, heuristic (cognitive shortcuts) bias occurred when trying to remember the magnitude and serial position of sequential items, in situations when the number of bits being processed were higher than the maximum information storage capacity of the participants could allow for (Stewart et al., 2005).

Shannon (1948) referred to entropy as an information measure for the amount of uncertainty in a non-deterministic situation. Using Shannon’s entropy, the source coding theorem can be derived which measures how noisy a given channel of communication is, i.e., how much of the information in the communication of the signal will be lost. This entropy *H*, can be defined as following:


(1) $$H\left(X\right)=-\sum_{i=1}^{n}P\left(x_{i}\right)logP\left(x_{i}\right)$$

Where given a discrete random variable *X* (e.g., a coin toss), with possible outcomes *x*_1_, …, *x*_*n*_, which have the probabilities of occurrence, *P*(*x*_1_), …, *P*(*x*_*n*_). Σ denotes the sum over the variable’s possible values and *log* is the chosen logarithm. A logarithm of base 2 gives the unit of bits and base 10 gives natural numbers.

So, any channel of communication, including a PH message, can be quantified in such a way, such as a telephone wire signal, internet signal, and even the cognitive system as a signal for communication, and all sources of signals are imperfect (lossy and not lossless) (Shamai et al., 1998). Indeed, early cognitive psychologists such as Miller (1956) demonstrated, through Shannon’s information theory, the limitations of short term memory, and that cognition was a lossy system for communicating information. This limitation, comes from a storage capacity limitation, in the same way as suggested by Stewart et al. (2005), it has been shown that when participants are asked to remember a sequence of information, then memory could store between 5 and 9 bits of information at any one time.

The cognitive short cuts present themselves as heuristics, which are imperfect tools of the cognitive system and can help process complex information in some situations, but can also cause systematic errors in decision making (Tversky and Kahneman, 1974; Redelmeier, 2005). One example of a heuristic is schema formations (Ferrario, 2002; McVee et al., 2005) which organize noisy information patterns of the world into categories which have similar components. For example, one schema could be a category of information about European people, and another could be about people from the United States (e.g., what they look like, how they behave, what language they might speak, what their political values are likely to be etc.). Crucially, a schema is a collection of information about objects, people, events and so forth.

Though there can be many benefits of these schemas in managing information about the world, and helping the individual make decisions, sometimes these schemas can lead to maladaptive thinking which can then lead to increased mental health disorders (Khadem et al., 2017), cognitive distortions (Mann and Beech, 2003), and even stereotyping and prejudice behavior (Fiske, 2000; Nelson, 2006). Specifically, in relation to PH messaging, such as the recent situation with COVID-19, it has been shown that due to the schema-based heuristics individuals use, this has led to bias, and ultimately in some situations, not adhering to following the guidelines (Madison et al., 2021).

Given the limitations of a schema based account of PH messaging, this paper will review the existing literature from behavioral economic (BE), heuristic, and behavioral analysis (BA) more deeply, in relation to explaining how cognitive biases in PH messaging occur and form, and how best to improve the effectiveness of PH messages. Traditional BE and heuristic theories are contrasted against more recent BA accounts, with some efforts given to how this may be developed into a mathematical model, which may be able to precisely define reinforcing and contextual effects of PH messages. This is given in the hope that they can provide an expanded viewpoint beyond BE and heuristic theories. Finally, it will then be argued that those involved in delivering important PH messages should be aware of the strengths and weakness of these different theoretical approaches, and utilize these approaches in order to effectively communicate PH messages to the communities they serve.

## Biased Decisions From Behavioral Economic Perspectives

Cognitive bias has been studied largely from a cognitive and a BE perspective, such as through exploring how the cognitive system perceives some value (gain) to a particular behavioral decision (called an expected utility), and some perceived loss for another behavior, in different situations. An early form of this gain-loss theory was Expected Utility theory, initially conceptualized by Von Neumann and Morgenstern (1947) and then further developed by Savage (1954). Expected Utility involves modeling (mathematically) how individuals make optimal (most gain) decisions under different levels of uncertainty, and can be expressed mathematically as in the following:


(2) $$U\left(A\right)=\sum_{o\in O}P_{A}\left(o\right)U\left(o\right),$$

Where *U*(*A*) is the expected utility of some action, *o* is a set of outcomes, *P*_*A*_(*o*) is the probability of outcome *o* conditional on *A*, whilst *U*(*o*) is the expected utility of *o*.

Expected Utility has been applied successfully to public policy areas such as in welfare politics to measure acceptability of mortality risks (Howard, 1968), also in health economics (Torrance and Feeny, 1989; Miyamoto, 1999), as well as effective altruism (Greaves and Pummer, 2019), utilitarian ethics (Mack, 2004), and outcomes of legal trials (Coughlan, 2000).

A later and highly influential cognitive bias theory within BE is prospect theory (Tversky and Kahneman, 1974, 1986; Kahneman and Tversky, 1979), for which Daniel Kahneman won the Nobel prize in economics. This theory assumes (unlike utility theory) that individuals are irrational agents. Prospect theory models how an individual makes actual (irrational) decisions under different levels of risk as opposed to optimal decisions as utility theory suggests. Within prospect theory there are two proposed distinct phases of cognition. The first is an editing phase called the framing effect, whereby the individual establishes a reference point through framing the information within the context of the environment. When faced with a situation which requires the individual to choose between two or more possible behavioral decisions, the context of the question can be presented (framed) in ways which highlight the situations’ positive (gains) or negative (losses) aspects for each possible decision. If presented in a more positive way (by highlighting gains) or negative way (by highlighting losses), then one decision can become more attractive than another. Crucially, this contextual presentation can drastically bias the way in which individuals’ perceive the value of the gains and losses in a particular environment.

In an example of this framing effect (Tversky and Kahneman, 1974, 1981, 1986), consider the following study (Tversky and Kahneman, 1981) where it was demonstrated that in some situations an individual’s decision making violates utility theory, and is more consistent with prospect theory: In a sample of 152 participants, they were asked to decide between two treatments for 600 people infected by a deadly disease. Two frames of reference (conditions) were presented to participants, a positive contextual frame and negative contextual frame. For the positive frame, treatment A was said it would save 200 peoples’ lives, whilst for the negative frame it was said treatment A would lead to 400 peoples deaths. For the positive frame of treatment B, it was said that there was a 33% chance of saving all 600 people and a 66% chance that it would not save anyone. For the negative frame of treatment B, it was said that there was a 33% chance that no one would die, and a 66% chance that 600 people would die. The researchers found that treatment A was chosen by 72% of participants vs. treatment B which was chosen by 28% of participants when presented within the positive frame (i.e., it will save 200 lives vs. 33% chance of saving all 600 people and a 66% chance that it would not save anyone), but this dropped to 22% for treatment A and increased to 78% for treatment B when frame in the negative way (i.e., 400 people will die vs. 33% chance that no one will die and a 66% chance that everyone will die).

This framing effect can be mathematically modeled through the second phase of prospect theory, which is the is called the evaluation phase (or decision-making phase), and in its simplest form can be defined mathematically as follows:


(3) $$V=\sum_{i=1}^{n}\pi \left(p_{i}\right)v\left(x_{i}\right),$$

**FIGURE 1:**
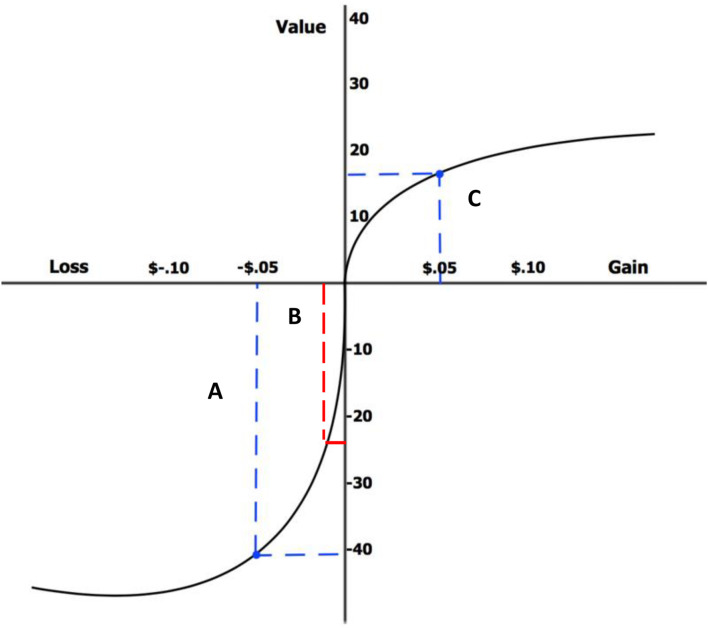
S-curve value function passing through the reference point. Image source (Laurenrosenberger, with permission to use and transform for any purpose) https://en.wikipedia.org/wiki/Prospect_theory#/media/File:Loss_Aversion.png.

Where *V* is the overall expected utility of the outcomes of some decisions (or prospects) *x*_1_, *x*_2_, …, *x*_*n*_ some individual makes, *p*_1_, *p*_2_, …, *p*_*n*_ are the respective outcome probabilities of occurrence for these decisions. π is a probability weighting function which captures a cognitive bias found in these types of studies. This specifically reflects that individuals tend to overreact to small probability events and underreact to larger probability events. *v* is a function that assigns a value to the outcome of a decision, which determines how painful or satisfying a decision will be. The value function in prospect theory is S-shaped, and this passes through the reference point (see Figure 1). This theory assumes that individuals are irrational (Tversky and Kahneman, 1974, 1986), as losses have been found to be more painful than gains feel satisfying, which means individuals are typically loss aversive.

Through the S-curve value function of Figure 1, different deviations from the reference point can be seen to be more painful or satisfying than others. For gains, the curve is concave and shallow, whilst for losses the curve is convex and much steeper. Therefore, a single unit deviation from the reference point will have a greater effect on decisions which involve loss than the same unit deviation from the reference for gain. Take, for example, the red line “B” in Figure 1, in the loss section of the graph, which shows a very steep curve in the value function, compared to the blue line “A” as it levels off. These are both much more steep than blue line “C” in the gain section of the graph, indicating that indeed this is a loss aversive function of reward.

There have been many examples of prospect theory being used successfully in the modeling of decision making for a variety of PH care settings. For example, it has been used to model health behaviors such as screening for disease (Schwartz et al., 2008; Adonis et al., 2015), the valuation of health (Moffett and Suarez-Almazor, 2005), health economics (Rice, 2013), and risk attitudes of people with chronic disease (Rouyard et al., 2018).

Prospect theory has also been utilized in PH communication settings by manipulating the framing effects of the message itself (Harrington and Kerr, 2017). Here, a PH prompt was used whereby the message stated that a transmittable disease was either deadly (condition 1) or easily curable (condition 2), and the response option was gain vs. loss (assuming the S-value function). The participants had to decide which of two outcomes they preferred, i.e., a certain low-risk (but potential for low reduction to severity) or uncertain high-risk (but potential for high reduction to severity) outcome. Participants tended to prefer certain low risk outcomes in the gain framed conditions, despite any consequence to severity of the disease (how impactful the outcome was), and no preference for the loss framed conditions.

Prospect theory was also used to explain stockpiling behavior during COVID-19. It was suggested that if individuals perceive the future as a period of scarcity and leading to a future scenario where it would be risky to identify essential supplies, then risk (loss) aversion could explain this protective behavior, i.e., to avoid future loss of supplies (Micalizzi et al., 2021). The theory also explains why compliance with social distancing measures reduce if PH guidelines state that individuals must isolation for a longer period than initially stated. Here it is assumed that expectations serve as the reference point, which means that an unexpected extension in isolation is perceived as a loss (i.e., a loss in social connection). The lack of compliance with the extended isolation guidelines is therefore the behavioral aversion to this loss (Briscese et al., 2020). An updated version of prospect theory called accumulative prospect theory (Tversky and Kahneman, 1992) has been used to demonstrate why populations conformed to PH rules initially, but then this conformity behavior decreases over time. This is explained, as individuals have a tendency for diminished sensitivity to loss as similar events accumulate. This means that people experience a reduced sense of pain for loss during the COVID-19 pandemic as the pandemic gradually spread and for a longer period (Ikeda et al., 2020; McCleskey and Gruda, 2021).

In terms of PH messaging specifically, smoking behavior has been shown to decrease after promoting smoking cessation through gain-framed messages (compared to loss-framed messages) (Toll et al., 2014). For example, gained framed PH message such as “quitting smoking will benefit your health by preventing lung and other cancers, heart disease, and stroke” were more effective at promoting smoking cessation than loss-framed messages such as “smoking will harm your health by causing problems like lung and other cancers, heart disease, and stoke.” Gain-framed PH messages (compared to loss-frames messages) also increased more vigorous exercise (Jones et al., 2003; Latimer et al., 2008), as well as improved diet and cancer prevention-related behavior (Satia et al., 2010).

So, clearly, understanding framing effects in PH messaging is important for improving adherence, and is particularly important when considering messaging where loss of life can be avoided, such as during the COVID-19 pandemic. However, prospect theory only describes loss-aversion framing effects, and there are many other cognitive processes relevant to ensuring effective PH messaging that cannot be explained by this simple BE account of prospect theory, for example, in the many situations which utilize heuristics in cognitive processing.

## Biased Decisions From Heuristics of the Cognitive System Perspectives

Some have referred to heuristics (Simon, 1990; Lau and Redlawsk, 2001) as useful strategies (i.e., through creating cognitive shortcuts) to cope with intractable problems, such as when playing a complex game such as chess, or making complex political decisions with many moving parts. This can also include our ability to form cognitive schemas (Ferrario, 2002; McVee et al., 2005) to minimize the amount of information the brain needs to process information. These types of heuristics are considered a decision strategy which enables fast, and economic decisions, by enabling the cognitive system to ignore less important information, or when time and information is limited, and this is considered crucial for adaptive behavior (Gigerenzer et al., 2011).

**FIGURE 2:**
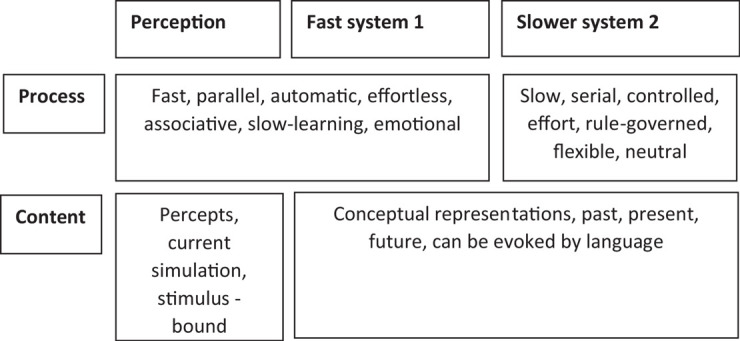
The two cognitive systems of fast and slow systems proposed by Kahneman. Image has been redrawn and adapted from Tagliabue et al. (2019) open access, permission grated.

**FIGURE 3:**
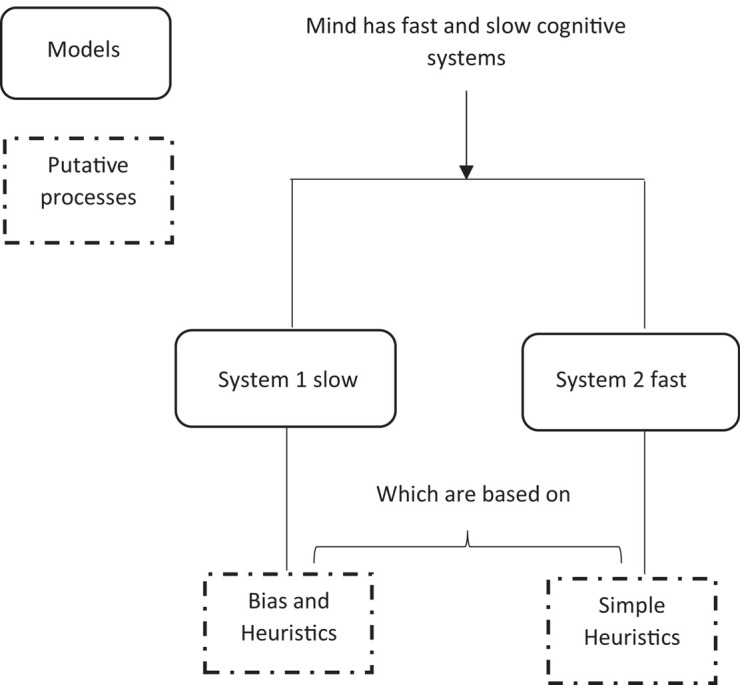
A simplified schematic representation of the Kahneman’s two system framework cognition. Image has been redrawn and adapted from Tagliabue et al. (2019), open accesses, permission granted.

Another example of heuristics within cognition, suggested by Kahneman (2011) describes a dual-process model of two distinct cognitive systems for processing information (see Figure 2 for the process of each system and Figure 3 for a simplified representation in a flow diagram form). This model is an attempt by Kahneman to explain why people sometimes act against their own self-interest, through the speed of responding of one system.

The first system of Kahneman’s (2011) dual-process model is described as a fast, automatic thinking system, which is based on emotions, vivid imagery, associative memories, and is susceptible to environmental influences. The second system is described as slower, and a more reflective system of thinking, which takes into account the goals and intentions of the individual decision maker. Kahneman (2011) suggests that when there are time demands or the information is too complex, then the fast automatic system takes over, and this can lead to biases in decision making.

However, as Kahneman (2011) pointed out, despite the increased potential for basic survival that heuristics such as fast thinking allows, this fast thinking heuristic can also lead to cognitive bias and ultimately decision errors. This is why the slower, more reflective second cognitive system is also important in many situations. Kahneman cites 20 cases where observed behavior deviates from correct answers which demonstrate cognitive bias. Below is just one such example (called the taxicab example) cited by Kahneman (2011), whereby individuals do not use all of the information given when deciding probabilities of outcomes, and this is attributed to the error of the fast (low effort) component of the cognitive system. Consider, for example, the following scenario and question: “A cab was involved in a hit-and-run accident at night. Two cab companies, the Green and the Blue operate in the city. You are given the following data: 85% of the cabs in the city are Green and 15% are Blue. A witness identified the cab as Blue. The court tested the reliability of the witness under the circumstances that existed on the night of the accident and concluded that the witness correctly identified each one of the two colors 80% of the time and failed 20% of the time. What is the probability that the cab involved in the accident was Blue rather than Green?”

**FIGURE 4:**
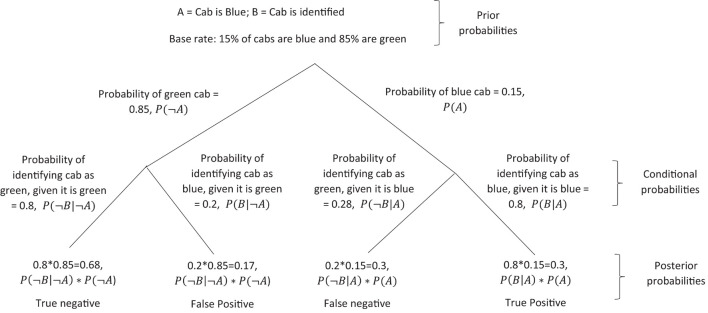
Bayes decision tree highlighting how cognitive bias can occurs with heuristics.

Kahneman (2011) suggested that this information can be combined by using Bayes theorem, which can be denoted in its standard form:


(4) $$P\left(A|B\right)=\frac{P\left(B|A\right)*P\left(A\right)}{P\left(B\right)},$$

where the probability of event *A* given some data *B* is equal to the probability of some data *B* given event *A*, multiplied by the probability of event *A* and divided by the probability of some data *B*. Given this theory, Kahneman suggested that in order to calculate what is the probability that the cab in the accident was really blue (event *A*), given that it was identified as blue (data *B*), then the information should be broken up into its types of information, where this information is taken as the posterior probability *P*(*A*|*B*) (see Figure 4). The true positive probability *P*(*A*|*B*)*P(*A*) divided by the probability of the cab being identified as blue *P*(*B*). Is the sum of the true positive and false probabilities, and can be denoted as:


(5) P(B|A)*P(A)+P(B|¬A)*P(¬A),

So, the correct answer to the question is 41% (see Figure 4 for step 3), however, Kahneman suggested that most participants incorrectly answered this question by responding with 80% as they do not process the prior probability that 15% of cabs in the city are Blue. The calculation can be followed below:


$$=\frac{P\left(B|A\right)*P\left(A\right)}{P\left(B|A\right)*P\left(A\right)+P\left(B|\neg A\right)*P\left(\neg A\right)},$$

=0.8*0.15(0.8*0.15)+(0.2*0.85),=0.120.12+0.17,=0.41(or 41%)

This of course could easily be applied to explain why biases occur in PH messages of today. Consider the recent COVID-19 crisis, given some fictional message which states the following: “In early March, 1% of the population of the country has been infected. An antibody test called a Lateral Flow Test (LFT) has been created and research has shown that if a person has antibodies (i.e., they are infected by COVID-19) the LFT will detect these antibodies 99% of the time, and give a negative result 1% of the time. If an individual does not have antibodies (i.e., they are not infected by COVID-19), then the LFT will test positive 99% of the time and negative 1% of the time.”

Given this PH message, the individual may want to decide what is the probability that they have antibodies (i.e., are infected by COVID-19), given they have received a positive test result. An individual may incorrectly assume that as the LFT will give a positive result 99% of the time when a person has antibodies, then they will have a 99% chance of having COVID-19 given a positive test. Again, if an individual decides this way, just like in the taxicab example, the individual is failing to process the prior probability given the population prevalence due to their fast, biased, heuristically based cognitive system.

However, like in the decision tree of Figure 4, the correct way to calculate this according Kahneman using Bayes theorem (Equation 4) is to multiply the sensitivity of the LFT *P*(*A*|*B*) (99% sensitive) and the prevalence of population which is infected *P*(*A*) (1%) to give the true positive probability *P*(*A*|*B*)*P(*A*), then divide this by *P*(*B*), which can be defined as sum of the true positives (sensitivity × prevalence; 99% × 1%) in addition to the false positives ((1 – prevalence) × (1 – specificty)). This calculation can be given as the following:


$$=\frac{P\left(B|A\right)*P\left(A\right)}{P\left(B|A\right)*P\left(A\right)+P\left(B|\neg A\right)*P\left(\neg A\right)},$$

$$=\frac{0.1*0.1}{\left(0.1*0.1\right)+\left(\left(1-0.1\right)*1-0.99\right))},$$

=0.1(0.1)+((0.9)*(0.1)),=0.10.1+0.9,=0.10.19=0.5(or 50%)

So, the correct answer of the probability of actually having COVID-19 given a positive result from the LFT is 50% (as give in the above calculation) and not 99% as may be incorrectly decided given a biased heuristic. These types of findings may lead to the assumption that perhaps PH agencies should be careful about how information is presented. It also may explain why there was mass indiscriminate antibody testing early in the pandemic, as the heuristic biases regarding the effectiveness of such testing would have been perceived as much higher than it actually was and therefore there would have been overconfidence in its success as an approach to controlling the virus. However, in the early stages of the pandemic there would have been a large number of false positives. More targeted testing on those who had symptoms or were in close proximity to others who had symptoms would have reduced the number of false positives, and possibly provided less disruption in the movement and activity of the public.

Unfortunately, this problem of how to promote effective PH messaging becomes even more complicated, as despite the great influence of Kahneman’s work, criticisms of these Bayesian type accounts of biases based on heuristics have been made. For example, some have argued that cognitive science has moved on from these early Bayesian accounts (Fiedler and von Sydow, 2015), and Pearl (2009) highlighted many limitations with probability theory when it comes to expressing causal assumptions. Some specific examples included criticisms over Kahneman’s (2011) “hot hand” in basketball heuristic example which has been shown to be a biased assumption in itself (Green and Zwiebel, 2013; Miller and Sanjurjo, 2015), also his findings on priming effects which did not replicate (Open Science Collaboration, 2015) and relied on some underpowered studies (Rivers and Sherman, 2018).

Streeb et al. (2018) further noted some of the problems with the way the data is heuristically interpreted, which can affect the way in which the data is visualized (e.g., global vs. local levels), and this is not accounted for by Kahneman. This relates to PH messaging which offer statistical analysis typically at the level of the global domain, but interpretation of this global data is often incorrectly made on the local (some sub-sample of the data) domain, which is relevant to local experience and knowledge, and can lead to local biases. For example, individuals may not abide by COVID-19 movement restrictions if they feel that locally the spread of the virus is not as prevalent as in other regions of the country (or at the global level) for which the message is directed to. In contrast, in some situations, local information can also be important, for instance, when deciding whether the COVID-19 vaccine is effective, based on which type of mutant form of the virus is present locally, and how effective a vaccine is for this particular strain of the virus, which requires local data sampling and analysis.

So, maximum payoff should be carefully chosen when considering using global or local statistics, and this may be entirely context dependent. Both Kahneman (2011) and Gigerenzer et al. (2011) agree that more information does not necessarily lead to better decisions, but instead better decisions are likely to be guided by more appropriate information at the global or local level. Given these many inconsistencies, and ongoing criticisms of cognitive interpretations of heuristics and bias, as well as the suggested need for more contextualized information, perhaps a broader account and framework is required to understand how to improve the effectiveness and compliance with PH messages. One model which maybe be useful in providing this contextual information is BA, and this may be helpful in offering an alternative perspective of why biases occur, and how best PH messages can be improved through such an approach.

## Biased Decisions From a Behavioral Analysis Perspective

A BA account can expand on BE accounts, as it can specify the broader contextual and functional properties which lead to biases in response to PH messages. BA and its contemporary extension contextual behavioral science (CBS) has a long tradition for developing conceptual and empirical studies which explain behavior acquisition and modification (Blackledge, 2003; Pierce and Cheney, 2004; Tagliabue et al., 2019). This approach includes a unique functional perspective which may be useful to understand more broadly how and why individuals behave given some PH message.

The unit of analysis in BA is the operant, which is a class of behavior modifiable by the direct contingencies of reinforcement and punishment that can alter the occurrence and variation of behavior within this class (Tagliabue et al., 2019). This contrasts which accounts which focus on a specific cognitive event as explained in the literature of heuristics such as by Kahneman (2011). BA refers to the direct contingencies of reinforcement and punishment that encompass antecedents (contingency) and consequences (reinforcement and punishment). From an experimental perspective, these are considered the independent variables which modulate the occurrence and variation of behavior. So, BA may give a unique perspective to determining the effectiveness of PH messaging by extending (or offering an alternative perspective to) Kahneman’s (2011) fast and slow thinking theory, from its own perspective of functional analysis. One specific example of this may be found with how PH messages act to modify behavior. Rather than focusing on the limited information processing problems of heuristics which have received criticism, BA can offer an interpretation based on how the PH message targets certain behavior through reinforcement or punishment, in order to achieve the desired behavioral modification.

One specific example of this attempt at behavior modification may be found in relation to nudge theory, which is a political science theory of how best to promote certain behaviors including those in relation to PH messaging, and can be understood through BA for behavior change (Kosters and Van der Heijden, 2015; Sunstein and Reisch, 2017; Simon and Tagliabue, 2018). This specifically refers to the promotion of positive reinforcement and indirect suggestion to influence the behavior and decision making of groups or individuals, which contrasts with other attempts to achieve compliance through direct coercion (negative reinforcement) such as legislation, and legal enforcement.

In some situations, nudges have been communicated within PH messages specifically to minimize risk to the public through nudging their decision making. In one example of this, patients risk awareness and decisions relating to living with cancer were altered when the PH nudge communicated the risk rate as “a 90% survival rate” compared to “a 10% mortality rate” (McNeil et al., 1982). In the first instance the patients became more likely to choose a suggested treatment option (e.g., survey over radiation therapy) when it was positively framed.

This nudging effect in relation to decision making when living with cancer, of course can be explained by framing effects of prospect theory (Kahneman and Tversky, 1979). However, from a BA perspective, nudges are perceived beyond simple framing effects and are, instead, assumed to be antecedents (which are presented by the environment) of target behaviors and the associated consequences of the resultant behaviors (Simon and Tagliabue, 2018; Tagliabue and Sandaker, 2019; Tagliabue et al., 2019). These antecedent nudges can come in many forms from the environment, such as company advertisements, economic messages, as well as Government PH messaging. Antecedents, or nudges, from this perspective, represent the starting conditions for which decisions can be made and for which lead to consequential behavior as a result of these decisions. This consequential behavior, in accordance with BA, can be reinforcing or punishing, and therefore the associated reinforcement or punishment can increase or decrease the probability of future occurrence of this behavior and related decisions, under similar conditions. Therefore, through this BA framework, nudges can be considered “natural” when they do not seek to alter behavior in a particular direction, or “contrived” when they directly seek to alter behavior in a particular direction, i.e., purposeful manipulation to bring behavior under the control of long-term and abstract reinforcement contingencies (Rachlin, 2015; Simon and Tagliabue, 2018).

The typical cognitive idea behind nudge is that changes in decision making is dependent on some influence of some aspect of the environment, such as how a PH message is framed (Saghai, 2013; Parkinson et al., 2014), so that automatic cognitive processes trigger a particular behavioral outcome. However, from a BA perspective, Tagliabue et al. (2019) have suggested that there should be greater focus on context interdependency which is an important feature of nudge theory from the BA perspective. This may be understood by exploring the two forms of behavioral shaping as long established in the BA literature (Skinner, 1966). The first is contingency-shaped behavior, which is the gradual shaping of behavior, such as learning to catch a ball, or walking, swimming, through trial and error. The other is rule-governed behavior or verbal behavior (Vargas, 1988), whereby a verbally competent human, can derive rules without having direct reinforcing experience with some event. For example, an individual does not need to touch a hot stove in order to experience a negative reinforcer (i.e., pain), they can simply learn by some verbal rule given to them by parents or some significant other, not to touch the hot stove because it can cause pain.

Three functional classes of rule-governed behavior in BA have been proposed, which include pliance, tracking, and augmenting (Zettle and Hayes, 1982). Pliance refers to rule-governed behavior which is controlled by speaker (socially) mediated consequences which corresponds a rule with some behavior. For example, if teacher said to a child: “you can only have playtime tomorrow if you complete all of your schoolwork today”, here, the behavioral act of completing schoolwork is under the control of the speaker mediated consequence (rule) of being able to have playtime tomorrow. It is perhaps important to note that the consequence does not need to be explicitly stated, as it can also be indirectly implied so as long as recipient of the message can infer and thus understand what the consequence of some given behavior will be. Though following this rule can have some benefit to the recipient who following it, this type of rule following can also become problematic when generalized, as it often does at some point during childhood (Ruiz et al., 2019). For example, it can lead to insensitivity to direct contingencies from the environment and other sources of stimulus control, thus lead to rigid and inflexible rule governed behavior (Törneke et al., 2008; Villatte et al., 2015).

Another functional form of pliance is called counterpliance (Hayes et al., 1989; Törneke et al., 2008), which is rule governed behavior under the control of socially-mediated consequences whereby the recipient of the rule will behave in a way that is different from the behavior reinforced by the rule. This form of pliance can also become problematic when generalized in a similar way to pliance, as it can desensitize the effect of direct reinforcing environmental contingencies (Villatte et al., 2015). Being sensitive to these direct contingencies is important, as they state the if-then conditions for which consequences occur given some given behavior, and within a particular context. Rule following which is harmful to the individual (e.g., such as opposing a social distancing rule when doing so causes spread of the virus) may arise in some situations if these direct contingencies are desensitized, and can lead to public harm or even death.

Tracking refers to rule-governed behavior which is predominantly controlled by the correspondence between the environmental contingencies and the rule (Harte and Barnes-Holmes, 2021). So, this type of rule puts behavior under the mediated control of the consequences specified (implicitly or explicitly) in the given rule, as the individual connects with (or tracks) these consequences by inferring the outcomes (consequences) of their behavior. For example, if the parent says to their child, “eat all your vegetables and you will be full of energy,” if the child successfully tracks and infers the consequence “full of energy” as positive and rewarding, such as having more energy to play, then the rule will be more likely followed.

Augmenting (Zettle and Hayes, 1982; Harte and Barnes-Holmes, 2021) refers to rule-governed behavior which can occur alongside pliance or tracking in a way which alters the extent to which a rule’s specified consequences for a behavior have reinforcing or punishing properties. Augmentals can take two forms: (1) motivative augmentals—this is where the established consequence functions as a reinforcer or punisher are temporarily increased or decreased. For example, if a speaker told a friend, “Let’s go for a jog now, it’s nice and warm outside right now,” and this temporarily increased the reinforcing value of jogging for that moment as it is warm in the present moment, it would be considered a motivative augmental and (2) formative augmentals—where a previous neutral stimulus established reinforcing or punishing consequence functions. For example, if you saw a small piece of metal on the floor and thought it was worthless, but a friend said, “that’s actually a very rare coin, and is worth a lot of money,” the previously neural stimuli (the coin) now has some reinforcing value, and you would now be more likely to pick it up.

Essentially, pliance, tracking, and augmentals are defined as antecedent verbal stimuli (Harte and Barnes-Holmes, 2021), as they all can influence the behavior of the receiver as they refer to (either explicitly or implicitly) apparent consequences of some suggested behavior, through actualizing specific functions in the stimuli which the receiver then behaviorally responds to. So, within the context of nudges, especially a contrived nudge, from a BA perspective, this can be thought of as some verbal or written rules which can govern and shape behavior deliberately.

The distinction between contingency-based and verbally governed rules has been suggested as non-trivial (Tagliabue et al., 2019). Through exploring their functional property differences, intriguing clues can be identified which are relevant to the analysis of bias and nudging, and particularly relevant within the context of PH messaging. One example of this, is that the natural nudges are context sensitive (i.e., they are sensitive to the environmental changes). However, in contrast to this, verbal rule-governed behavior may not be sensitive to these environmental changes, and instead can be fixed despite changes in context (Hayes et al., 2009).

This lack of context sensitivity can have very damaging and detrimental effects to the individuals who are controlled by rules. As human beings who have the capacity for language, individuals can pursue rules even when they lead to an adverse outcome, just because they are deemed as “correct.” An example of this could be a rule whereby a parent told you to “eat genetically modified (GM) foods because they are better for you than organic food because fewer pesticides are used on them.” If you followed this rule despite growing evidence suggesting that GM foods are overall less healthy compared to organic foods, you are insensitive to the changing context of the evidence. By ignoring the evidence, you would be putting your body at greater risk of ill health.

Ultimately, though BA can give some important insights into rigid rule following, such as its specification of pliance, tracking, and augmentals which can have applications to facilitating nudges in PH messages, in the form of strengthening the reinforcing value of the message (such as through motivational augmentals), it has been suggested that this analysis alone is still limited. Specifically, it is suggested that these terms are not precise enough to be considered technical terms with a high degree of predictability of outcomes (Harte and Barnes-Holmes, 2021), and should be considered middle-level (simpler and less clearly defined) clinical terms. As such, as Harte and Barnes-Holmes suggest that a broader and more technical account should be assumed which expands the BA framework by considering broader contextual relations to explain why rules are followed in some situations and not others. This broader contextual and relational framework of this BA approach may be advantageous in order to increase descriptive and predictive power of this model in order to explain and predict the effectiveness of PH messages.

## A Contextual Extension to the Behavioral Analytical Perspective of Biased Decisions

The BA CBS extension of this approach is called relational frame theory (RFT) (Barnes-Holmes et al., 2001; Torneke, 2010) and is a post-Skinnerian behavioral account of language and cognition. This naturally expands its BA foundation to give a functional contextual ontological account and seeks to explain and model human cognition and language. It does this through conceptualizing language and cognition in terms of relational (operant) responding, whereby stimuli are connected to other stimuli, and a response is given as a function of one stimulus in connection with another stimulus or other stimuli. One class of contextually controlled responses are called derived relational responding (DRR) which can either be arbitrary or non-arbitrary (Barnes-Holmes et al., 2001; Whelan and Barnes-Holmes, 2004; Levin and Hayes, 2009; Torneke, 2010; Campbell et al., 2011). Non-arbitrary relational responding (NARR) is based on physical features such as magnitudes of size, shape, or color of the stimuli involved, while arbitrarily applicable relational responding (AARR) is instead controlled by historical contextual learning and not stimulus physical properties.

RFT specifies several different patterns of AARR, for example (and not exclusively): co-ordination (e.g., stimulus X is the same as stimulus Y); comparison (e.g., an elephant is bigger than an ant); opposition (e.g., up is the opposite of down); distinction (e.g., you are not the same as me); hierarchy (e.g., a Seagull is a type of bird); and perspective-taking (referred to as a *deictic* relation) which involves the interpersonal (I vs. YOU), spatial (HERE vs. THERE) and temporal relations (NOW vs. THEN).

In addition to these patterns of AARR, RFT relies specifically on a history of operant conditioning across many situations in order for the patterns of relating to be established. The patterns of relating refer to the relational frame, which is comprised of three core properties: (1) In *mutual entailment* (ME), relating to one stimulus entails relating to a second stimulus. For example, if X is bigger than Y, you can derive (entail) then that Y must be bigger than X. If you are told X is the same Y, then you can derive that Y must be the same as X. (2) In *combinatorial entailment* (CE), relating a first stimulus to a second and relating the second to a third facilitates entailment between the first and the third stimuli. For example, if you are told that Tom is faster than Steve, and Steve is faster than Paul, then you can derive (combinatorally entail) that Tom must be faster than Paul, without having been directly told this. (3) In *transfer (or transformation) of stimulus function* (ToF), any function of a stimulus that is connected (related) to another stimulus or stimuli through a relational frame, may be transferred or transformed according to the relations that the stimulus shares with other stimuli also connected within that frame. For example, if you were given a shock every time you pressed a button called “button A,” and you were told that “button B” is greater than button A, your fear of pressing button B may be greater than pressing button A, even when the shock was directly paired with pressing button A and not pressing button B. This occurs because fear as a behavioral function that is established to pressing button A is transferred to pressing button B, and in greater magnitude. So, the fear function transformed (increased) because of the comparison relation (being told button B was greater than button A) between A and B.

From the BA perspective previously mentioned, rule-governed behavior was assumed to relate to some verbal variable which places the behavior of the individual under this verbal rule-governed control (through pliance, tracking, or augmentals). In a case whereby there was a non-verbal operant, then it was understood that the behavior of the individual was contingency-shaped, i.e., modified in line with the consequences of such behavior. RFT (Barnes-Holmes et al., 2001; Torneke, 2010) takes this one step further by assuming that learning occurs in a complex network of relations, where language can allow for the changing of the symbol’s (nouns, sentences, and symbolic relations) function through ToF, which can lead to the individual experiencing different adverse (punishing) or rewarding consequences based on these networks of relations.

From this RFT perspective of rule-governed behavior, humans as verbal organisms can learn through AARR any arbitrary rule, for example, they can learn that a five-cent coin is worth less than a 10-cent coin despite the fact that the five-cent coin is physically larger than the 10-cent coin in physical size (Hayes et al., 2003; Vilardaga et al., 2007). DRR of stimuli within the network of relational frames allows the verbal organisms to relate stimuli in an almost infinite amount of ways, regardless of any history of direct learning through reinforcement for relating the stimuli in a specific way (Blackledge, 2003).

**FIGURE 5:**
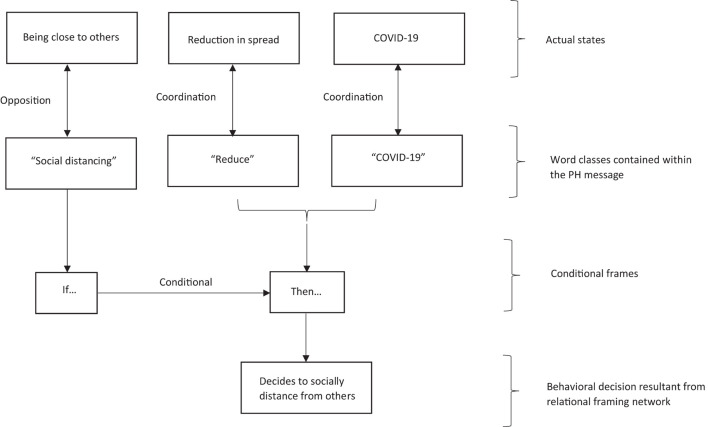
A schematic representation of a relational framing network formed from reading a PH social distancing message, as suggested by the standard RFT model. Figure is redrawn and adapted from Stapleton (2020), permission granted from Elsevier.

In terms of an RFT interpretation of PH messaging, consider the following example, whereby a PH message states that everyone needs to be at least one meter distance from others outside of their household, and at all times, to prevent the spread of COVID-19. According to an RFT interpretation, then this rule-based information (stimuli) are organized into a network of relational frames (Stapleton, 2020; Stapleton and McHugh, 2020), whereby word classes (e.g., COVID-19) are framed in coordination (i.e., identifying sameness) with an event class (i.e., the actual COVID-19 virus), and framed in distinction to others (i.e., the act of distancing from others). What is interesting about the RFT account, is that it has a framework for explaining how the function of “distance with others” (distinction relation with others) changes through ToF, in that “distance with others” was neutral, and now given the PH message rule, individuals may be fearful for catching or spreading the virus (if they are not distanced with others) through the consequences of what danger the virus will bring (e.g., death, further long term lock down, etc.), and subsequently follow the rule by distancing from others (see Figure 5 for how the relational network may develop).

So, in relation to bias in PH messaging, the RFT perspective gives an interpretation of its context interdependency, which refers to the property of the operant for which is described as a learned (or reinforced) decision behavior, and is functionally interrelated through being influenced by, and having influence over, its environmental contingencies. Many of these environmental contingencies are social in nature, such as communicating, therefore can be particularly helpful in providing a broader reference model which goes beyond Kahneman’s (2011) think fast, think slow theory, or prospect theory (Tversky and Kahneman, 1981), as it can explain why nudges work in some cases and not others given this context interdependency.

From the Kahneman dual process (2011) framework, nudges are understood in terms of the type of system it targets, either the automated fast system or slower more conscious system. It has been suggested that natural nudges (which nudges behavior without any real attempt to do so), may utilize the automated system one, and contrived nudges (which have the intention to manipulate behavior more directly) rely on system two (Rachlin, 2015), as they activate more deliberate, reflective components of the cognitive system, which require more resources and time. However, Tagliabue et al. (2019) suggests that though Kahneman’s prospect theory (Tversky and Kahneman, 1974, 1986) and the dual-process heuristic model (Kahneman, 2011) offers a useful framework for affective forecasting in the form of modeling decision processes of loss aversion which deviate from normative pre-established values (utilities), as well as bias based on heuristics in processing, it does not account for very important deviations from selective and contextual contingencies. So, rather than explain through cognitive errors, RFT explains nudges in PH messages through AARR. This can have important advantages in explaining rule governed behavior and why individuals follow or deviate from these messages, compared to previous theories put forward by Kahneman and others. For example, for heuristics, RFT can explain these are behavioral responses which are under the control of relevant relational networks. Through modeling the conditions of which these reinforcers of consequences take place, this, through the RFT model, can give some clues how the public may respond to a PH message and beyond simple fast-slow heuristics, as it takes into account this context interdependency.

**FIGURE 6:**
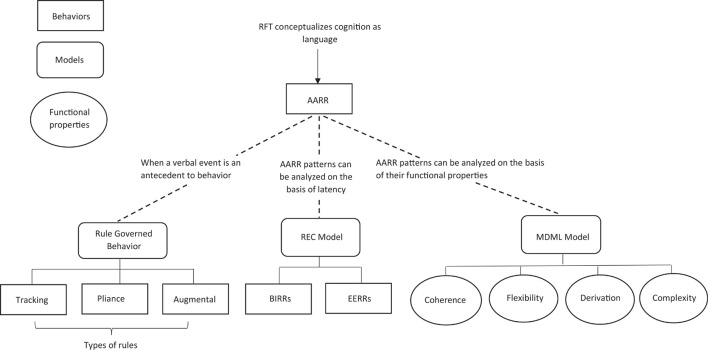
The adapted REC RFT framework of cognition which includes fast and slower responding. Image has been redrawn and adapted from Tagliabue et al. (2019), open access, permission to reuse and adapt granted.

One important extension to the RFT model, which is particularly important for modeling bias, is the relational elaboration and coherence (REC) model (Barnes-Holmes et al., 2010b; Hughes and Barnes-Holmes, 2013) (see Figure 6). This RFT-based account of cognition models the formation and retention of opinions, beliefs, decisions, prejudice, and bias. This extension, takes into consideration of fast and slow responding, in a similar way Kahneman’s (2011) dual-process model did, but again, it does this with the advantage of considering the reinforcing context for which consequence occurs, and how this information forms within a relational network (as was the case for the standard RFT approach highlighted in Figure 5).

The REC extension to RFT suggests that there are two speeds for responding, either brief and immediate relational responding (BIRR) or extended and elaborated relational responding (EERR), and these are in the form of operants which can interact with one another within a relation network (Barnes-Holmes et al., 2010a). BIRR refers to responses which occur within a few seconds of the stimuli event (antecedent), whilst EERR refer to responses which occur after a longer period of time (between stimuli and response) (Barnes-Holmes et al., 2010a).

The advantage of this approach is that it has been suggested (Tagliabue et al., 2019) that BIRR and EERR address processes which are included in the dual-process (fast and slow) of Kahneman (2011). This includes application with the associatively coherent model of Morewedge and Kahneman (2010), which suggests that when information being processed is coherent with existing knowledge that are activated quickly in system one and this can be implicit.

Here, the REC RFT model interpretation of associative coherence, is that being coherent is socially reinforced in childhood (Tagliabue et al., 2019) and continues in adulthood when it develops with language acquisition. For example, a parent or teacher will reinforce the correct and consistently coherent labeling if a light bulb, for example, when the child points at the light and labels it consistently this way they may be rewarded by the teacher and parent. So, coherence is reinforcing whilst uncertainty (or high informational entropy), which is relational incoherence, is not reinforced within society.

Some evidence (Prevedini et al., 2011; Villatte et al., 2015) has supported this suggestion of a preference for coherence, in the form of studies which have identified that individuals who tend to create coherent stories even when they are asked to describe inconsistencies in their experience, and even when describing a coherent story can lead to the cost of increased psychological suffering in a clinical setting. In addition to this, additional supportive evidence comes in the form that as fast processing is typically assumed to be largely implicit, then BIRR can be recorded using a relevant measure. RFT implicit measures such as the Implicit Relational Assessment Procedure (IRAP). This has be utilized to measure how fast relational responding takes place and has demonstrated (Barnes-Holmes et al., 2010a; Maloney and Barnes-Holmes, 2016; Maloney et al., 2020) its effectiveness in measuring the coherence of how uniform a given relational response is with the individual’s previous learned experiences.

In line with the assumptions of the REC RFT model, coherence may present itself within different topographies of AARR. For example, it may explain why you may trust someone with whom you have had previous positive experiences with. Similarly, it may explain why you decide to buy a particular brand because of a positive history of purchasing products of this brand (brand fidelity), or even why you decide to follow some PH message based on your belief that they it is enforced by science, and that you trust scientific opinion. Through REC RFT, these biases maybe understood within the context of being reinforced by coherent self (verbal) narrations. A simple instruction or nudge of “think the opposite” has not been shown to change such coherent behavior in IRAP tasks (Barnes-Holmes et al., 2010a). Similarly, with serotyping, it is suggested that this will depend on the coherence of the individual’s verbal history, as the first DRR in the form of BIRR may lead to more stereotyping (greater coherence with learned verbal stereotyping), whilst with less time constraints and more time to reflect, this may provoke DRR in the form of EERR (Roche et al., 2002), may lead to more complex and varied relations (less coherence with learned verbal stereotyping) which can be explored by the individual through reflection (such as through customs, traditions, etc.).

Within the context of effective PH messaging, there may be several reasons why some rules may not exert influence over behavior according to the REC RFT model. The model assumes that if a rule has components within the relational network which are contradictory and of low coherence to the network as a whole, then it is less likely to be followed (Törneke et al., 2008; Tagliabue et al., 2019; Stapleton, 2020). So, for example, if there is greater coherence to previous learned verbal histories, then there may be insensitivity to contingency effects in the changing environment as identified in cases of verbal-rule governed behavior (Tagliabue et al., 2019). For example, if close contact with others has been reinforced (and typically is) through socialization and pliance, then a PH message which is developed for the changing environment of COVID-19 which says “do the opposite of staying close (socially distance),” is likely to lead to some behavioral resistance, and this was certainly the case with the COVID-19 pandemic (Forsyth, 2020; Arli et al., 2021). In other situations whereby a PH message may not be adhered to, this may be due to the individuals believing that they do not have the skills required (low-self efficacy) to implement the rule, or that the source of the rule is deemed unreliable (Stewart et al., 2006).

So, in order to increase the effectiveness (behavioral adherence) of PH messaging, then it may be reasonable, given this REC RFT model, that the PH body such as the World Health Organization (WHO), or National local PH agency of Government, recognize the importance of acquiring some understanding about the general knowledge of the public, whether at the local or global level. This would allow the PH agency to address any perceived incoherence the public’s relational knowledge networks. This approach has been successful when, for example, attempting to increase the adherence to healthcare treatment plans on an individual level, whereby patient centered engagement is usefully applied to tackling problems with disengagement with such incoherent knowledge beliefs within their relational networks (Martin et al., 2005).

In addition to this, those with greater health literacy have been found to adhere more to PH messages when compared to those with lower health literacy (Cho et al., 2008; Miller, 2016). So, in a similar way to the previous example, PH agencies should therefore seek to understand the general knowledge and health literacy and self-efficacy of specific local regions, which maybe based on educational levels, socio-economic status, and other variables related to geo-location, as opposed to delivering a PH message at a global level as delivered by the WHO during the COVID-19 pandemic and other situations. So, more localized targeted PH messaging which are sensitive to the contextualized knowledge of local populations may improve adherence to a PH message as they become more coherent with local knowledge.

Some further interesting work has specifically explored modifying reward and punishment contingencies, and specifically how predictive change is dependent on the levels of mindfulness the participants have (O’Connor et al., 2019). Here, in contingency shifting phases, responses which were previously reinforced were no longer reinforced and responses which were not previously reinforced were now reinforced. Participants, during these shifting phases formed new strategies in order to gain the most reward. Again, this may be applicable to effective PH messaging, whereby adherence maybe increased when the messages are framed toward reward. This is a similar approach to how Kahneman’s framing effect of prosect theory had been adapted for PH messaging, however, here through the REC RFT approach, greater predictability of reinforcing contingencies can be made, and thus greater control and adherence over the targeted behavior through a PH message can be achieved, i.e., through contextualizing of local knowledge and thus maximizing coherence within the public’s relationally framed networks.

Motivative augmentals, through this RFT approach can expand on the simpler BA account, by suggesting that the PH message should be orientated to ensure the rule is connected with what matters (their values) to the targeted local population it is directed at in order to improve adherence. This should allow for some temporary (or even longer term) change in the reinforcing value of the consequences specified in the rule given (Ju and Hayes, 2008; Törneke et al., 2008; Villatte et al., 2015).

This motivative augmental was actually carried out quite effectively (Smith et al., 2020; Stevenson et al., 2020) in the United Kingdom, for example, where a simple message of “Stay home, protect the NHS, save Lives” (Department of Health and Social Care, 2021) was broadcasted daily on national television. The message being simple, was likely accessible to even those with low health literacy, therefore promoted high coherence within the publics relational networks, and was a rule framed to reinforce what most people cared about and valued (therefore utilizing motivate augmentals), i.e., saving lives and protecting the NHS. This also indirectly targeted broader values such as a sense of community, and common societal values, which is identified through tracking the correspondence between the environmental contingencies and the rule (Harte and Barnes-Holmes, 2021). Furthermore, this maybe further reinforced through local contextualized tracking, as societal community values means that self-sacrifice through isolation is both locally reinforced, which further strengthens the behavioral adherence to the rule, and could explain why this simple PH message was so successful.

For all of these reasons, it is clear that this REC RFT account expanded from BA, can be valuable for increasing the predictive power of effective PH messaging, and can be a useful addition to Kahneman’s prospect theory and dual process theories, by offering a much broader contextualized approach to how knowledge is relationally framed in a local and global population.

However, one main area that may further improve the predictive and descriptive power of these RFT-based approaches is through mathematically defining this model. Though there has been considerable working in defining basic BA, there has been little in the way of mathematically defining this adapted RFT model. It has been suggested (Shull, 1991; Myung and Pitt, 2002) that models which are not mathematically defined are useful to describe general trends, but are clumsy for describing precise relationships, whilst the language of mathematics can describe precisely and succinctly how terms are related to one another. This perhaps gives the model greater descriptive and predictive power to precisely define processes and outcomes, as seen in other areas such as medicine, biology, and neuroscience (Costanza and Sklar, 1985; Kaplan and Craver, 2011; Benzekry et al., 2014). This maybe particularly useful in modeling relational processes, context, and outcomes of the RFT approach which explain how bias occurs within the context of PH communication.

## Mathematical Considerations for Relational Frame Modeling of Risky Decisions

Though little effort has been made to define the more recent RFT adaptations of the BA approach, there have been considerable effort to define both classical and operant conditioning mathematically. So, perhaps the simplest starting point for some generalization of a mathematical approach for RFT, is to first simply identify the mathematical form for pavlovian strength of association. Pavlovian classical conditioning has been highlighted by Rescorla and Wagner (1972), to describe the strength in learned associations between the conditioned stimulus (CS) and the associated unconditioned stimulus (US) such as a bell through pairing. The strength of associations can thus be denoted as the following:


(6) $$\Delta V_{i}=\alpha_{i}\beta_{1}\left(\lambda -\sum_{keS}V_{k}\right),$$

Where α_*i*_ reflects the intensity or salience of CS_*i*_, β_1_ reflects the rate of learning on US trials, λ is the maximum possible level of association strength conditionable with that with that US intensity, and $\sum_{keS}V_{k}$ is the sum of the associative strengths between all of the CS and the US stimulus elements occurring on a given trial.

This could be usefully employed in situations when modeling non-verbal behavior, where language is not considered an essential component, or if the organism does not have language (Barnes-Holmes et al., 2001), in order to formulate strength of association given relevant conditioning parameters within the learning history and context (e.g., salience, learning rate).

However, RFT is a post-Skinnerian language model, so an RFT model for decision making requires mathematically defining operant conditioning. Two major theories about punishment referred to in the in the literature are the negative law effect (Rachlin and Herrnstein, 1969) and the avoidance theory of punishment (Dinsmoor, 1954, 1977). On the surface, these two theories say the same thing, i.e., that punishment will cause a decrease in the frequency punished behavior. However, once quantified in a mathematical model these both say quite different things. Deluty (1976) and De Villiers (1977, 1980) quantified these into models (avoidance theory of punishment and negative law effect, respectively). Herrnstein’s (1961) matching law begins in both of their mathematical models but then move into different directions.

The matching law, in its simplest form, can de denoted as follows:


(7) $$\frac{B_{1}}{B_{1}+B_{2}}=\frac{R_{1}}{R_{1}+R_{2}},$$

Where *B*_1_ and *B*_2_ are the rates of response on reinforcement schedules 1 and 2, and the rates of reinforcement on these schedules are denoted as *R*_1_ and *R*_2_. So, these have been usefully applied to situations of variable-interval (VI) schedules (frequency) of a reinforcer. For example, consider the following example, if pressing key one delivered 75 reinforcers per hour and pressing key two delivered 25 reinforcers per hour, then equation six predicts that the individual will press key one 75% of the time and key two 25% of the time.

To expand this equation further to include punishment whereby both keys begin to deliver punishers (for example, and electric shock) at a rate of 20 shocks per hour for each key, then according to De Villiers (1977), as reinforcement is the opposite of punishment as suggested by the negative law of effects then punishers delivered should be subtracted from the reinforcers given. This can be denoted as following, where *P*_1_ and *P*_2_ are the rates of punishment of the two keys:


(8) $$\frac{B_{1}}{B_{1}+B_{2}}=\frac{\left(R_{1}-P_{1}\right)}{\left(R_{1}-P_{1}\right)+\left(R_{2}-P_{2}\right)},$$

Deluty (1976) suggested that by punishing one response, this would actually increase the reinforcement for the other responses. However, experimental data with pigeons (De Villiers, 1980) have shown a preference for De Villiers (1977) equation seven. So, despite the two approaches by Deluty and de Villers starting from the same principles of reinforcement and punishment of the matching law, subtle differences in their mathematical interpretation lead to different predicted outcomes, which highlights the importance of selecting the model with the greatest predictive power, which accurately model the behavioral data (Mazur, 2006).

Another problem that had been addressed mathematically was how to model the decrease in the strength of a reinforcer with increasing delay from further reinforcement. One suggestion (Hull, 1943; Killeen, 1994) is that the gradient of delay-of-reinforcement can be described by an exponential function, given as:


(9) $$V=A_{e}^{\left(-KD\right)},$$

Where *V* is the value or reinforcing strength of a reinforcing consequence delivered after a delay of *D* seconds. *A* is the strength (or value) of the reinforcer when it was given immediately, *e* is the base of the natural logarithm, and *K* is a parameter which determines the rate to which *V* declines with increasing delay of further reinforcement.

It should also be noted that a hyperbolic version of the equation has also been suggested (Mazur, 1987), though the Killeen (1994) and Killeen and Sitomer (2003) exponential equation in generally preferred by behavioral economists (Mazur, 2006). This is because it successfully models data for temporal discounting tasks of monetary gain as well as self-controlled choice situations (i.e., changing one’s mind such as being on a diet but then deciding to eat more during a meal) (Green et al., 1994, 1997). The Killeen (1994) and Killeen and Sitomer (2003) decay function has been applied to data whereby pigeons choose between 45s of exposure in one situation, or VT schedules and even a single presentation of a delayed reinforcer, whereby the delay was adjusted over trials. The model accounted for 99% of the variability in the data, demonstrating the effectiveness of this model (Mazur, 2000).

In terms of PH messaging, this could be applied to modeling and predicting possible adherence to a PH message given different trade-offs between delay, rate, and strength of reinforcement given within the PH message. This can be done, as the Killeen (1994) and Killeen and Sitomer (2003) model specifically defines the value (or strength) or a reinforcer or reinforcing consequence, and models how this value decreases with any delay of further reinforcement. It could also be applied to modeling adherence of a PH message when given some motivative augmentals which have some temporal reinforcing benefit of the message. So, such applications could help PH agencies understand how frequent the reinforcing PH messages would need to be provided in order to maximize optimal compliance and adherence to the message both at global and local levels.

However, these are still simple models, and they do not model decisions about complex societal interactions, which often have schedules of reinforcers in chains depending on which decisions is made. Interfering with these interactions, to try to get the public to adhere to a set of PH message rules needs a mathematical definition of the linked schedules of reinforcers, strengths of reinforcers, identifying the effects of contradictory reinforcers, and any delays of reinforcers. To do this, one useful model within the behavior literature which could be applied to an RFT model, is the contextual choice model (CCM) (Grace, 1994), and this can be given as:


(10) $$\frac{B_{1}}{B_{2}}=\left(\frac{r_{i}1}{r_{i}2}\right){\left(\frac{r_{t}1}{r_{t}2}\right)}^{\left(T_{t}/T_{i}\right)},$$

Where *B*_1_ and *B*_2_ are response rates in the initial links of concurrent-chain schedule, and rates of reinforcement are expressed in the initial link as *r*_1_ and *r*_2_. *T*_*t*_/*T*_*i*_ is a ratio whereby *T*_*t*_ is the average terminal-link duration, and *T*_*i*_ is the average initial-link duration. As the ratio *T*_*t*_/*T*_*i*_ are exponents for the terminal link, this means that differences in these links will have a greater effect on behavioral decisions made when the links are longer as opposed to the length of duration at the initial links. Grace, who was guided by the work of Rachlin and Herrnstein (1969) proposed that when reinforcers differed along two or more dimensions (e.g., quality, rate, amount, delay), these different factors can be combined through multiplication to give a measure for overall reinforcement value.

In terms of an RFT account which can incorporate the Equations 7–10 through the matching law (Equation 7), punishment (Equation 8) delay-of-reinforcement (Equation 9), and chained-linked schedules of reinforcement (Equation 10). This can then be applied to a set theory mathematical account of the RFT approach for how information (stimuli) is relationally framed within the network. In order to mathematically define the components of ME, CE, and ToF with the framing of stimuli withing this model, set theory can be used to denote this. For example, equivalence from RFT can be stated as follows for equivalent relational properties between two sets using set theory (the three horizontal bars sign denotes equivalence):


(11) $$\left\{AR_{\text{x}}\text{B}\right\}\equiv \left\{AR_{\text{y}}\text{B}\right\}$$

However, if the symbol ||| is used to denote a shared relation (AND) within the set, as suggested in a previous study (Gilroy, 2015), then Equation 11 can be expressed more succinctly, in the form of ME. In the following example of ME, describing a black redback spider (A) as being “more dangerous” (R_*x*_) than a gray blueback spider (B) derives the relation through ME that, therefore, that a gray blueback spider (B) must be “less dangerous” (R_*y*_) than a black redback spider (A), whereby *C*_*rel*_ is denoting that there is a contextual relation within the set. ME can therefore be denoted as:


(12) $$C_{\text{rel}}\left\{AR_{\text{x}}\text{B}|||BR_{\text{y}}\text{A}\right\}$$

Or in plain English:

In the jungle (*C*_*rel*__)_…{a black redback spider (A) is “more dangerous” (R_*x*_) than a gray blueback spider (B) AND (|||) a gray blueback spider (B) is “less dangerous” (R_*y*_) than a black redback spider (A)}.

An additional condition can be included for CE, which can be denoted as:


(13) $$C_{\text{rel}}\left\{{\text{AR}}_{\text{x}}B\mathrm{and}BR_{\text{y}}\text{C}|||AR_{\text{p}}C\mathrm{and}CR_{\text{q}}\text{A}\right\}$$

Or in plain English:

In the jungle (*C*_*rel*__)_….{a black redback spider (A) is “more dangerous” (R_*x*_) than a gray blueback spider (B) and a gray blueback spider (B) is “more dangerous” (R_*y*_) than a green purpleback spider (C) AND (|||) therefore, a black redback spider (A) is “more dangerous” R_*p*_ than a green purpleback spider (C) and a green purpleback spider (C) is “less dangerous” (R_*q*_) than a black redback spider (A)}.

A further condition can be included to account for ToF, whereby ^*f*1^ is the function “fear,” can be denoted as:


(14) $$C_{\text{func}}\left[{\text{C}}_{\text{rel}}\left\{AR_{\text{x}}B\mathrm{and}{\mathrm{BR}}_{\text{y}}\text{C}\left\{{\text{B}}_{\text{Rp}}^{\mathrm{f1}}\mathrm{and}C_{\text{Rq}}^{\mathrm{f2}}\text{B}|||A^{\mathrm{f3}}\right\}\right\}\right]$$

Or in plain English:

*C*_*func*_—WHEN told dangerous black redback spider live IN the jungle.

C_*rel*_—WHILE talking to a snake specialist, and deciding whether to take an adventure trip to jungle or not.

Here → is used to denote the direction of a ToF from one stimuli to another.

Jungle (A) is “related to” (R_*x*_) you (B; as you decide to walk through the jungle) and you (B) are thus “related to” (R_*y*_) dangerous black redback spider (C; who you are told live in the jungle and may encounter one if you decide to walk through the jungle) THEN you (B) are “fearful” (share functional property of fear—^*f*1^_*Rp*_) of jungle (C→A ToF; as you have been told the spiders that live in the jungle are dangerous, so the fear of spider entails with the fear of jungle as you become afraid of the jungle) AND jungle (C→A ToF) is “feared by” (^*f*2^_*Rq*_) you (B; as the ToF is mutually entailed). This implies that the jungle (A) through ToF now has the function of fear (^*f*3^), and causes the feeling of fear when you think about walking through it.

In order to incorporate this into single model which may be useful for modeling likelihood of decisions being made when given a PH message, some reward function, the environment, and any chains of event reinforcers, a relatively straight forward approach would be to incorporate it into a Markov decision process (MDP) which is designed to model reinforcement learning (RL) (Sutton and Barto, 2018). This approach does not take into account any prior knowledge the individuals may have, and is has been usefully applied to modeling how equilibrium may arise in bounded rationality. This is specifically useful for modeling potential outcomes for decision making problems where there is uncertainty, and utilizes a very simple default architecture based on reinforcement learning, which can be adjusted specifically according to the RFT model.

The challenge of RL is to design a policy of what actions to take, give a state *s*, to maximize the chances of getting the greatest reward, hence it is an optimization problem (optimizing reward). What is the probability of taking action *a*, given that the individual is in state *s*? This is based on a probability as the environment is not deterministic as it can change, hence a probabilistic environment.

**FIGURE 7:**
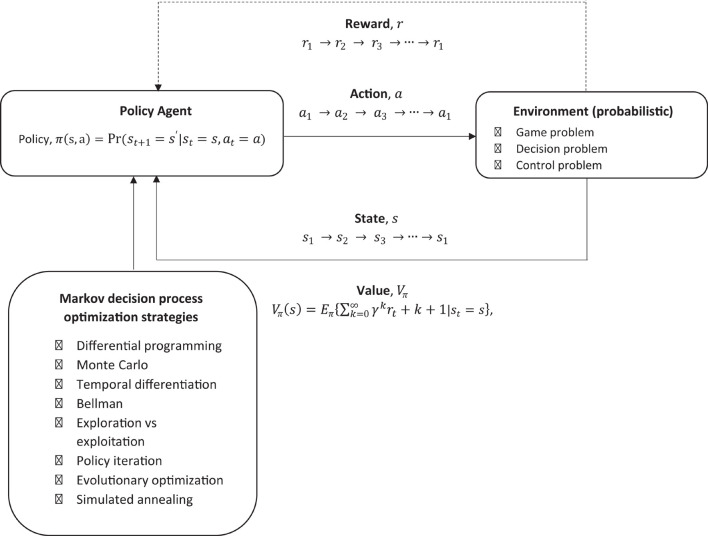
Overview of reinforcement learning machine learning framework which models decision making within an environment of reward.

In this approach (see Figure 7), basic reinforcement is modeled with a MDP, whereby the probability *P* given an action *a*, that a state *s* will transition into another state *s*′ can be given as:


(15) $$P_{a}\left(s,s^{\prime }\right),$$

At each step, the environment is in some state *s*, and the individual will make some decision to take some action *a* which is available is that state. This action transitions the state *s* into a new state *s*′, and the individual can then be rewarded *R* for this action *a* which led to this new state *s*′, and, therefore, *R*_*a*_ is the reward (or expected reward) of transitioning from the current state *s* and into state *s*′ as given in Equation 16 below:


(16) $$R_{a}\left(s,s^{\prime }\right),$$

So, this Markov decision process is a four component tuple (*S*, *A*, *P*_*a*_, *R*_*a*_), where *S* is a set of states called the state space, *A* is a set of actions called the action space, *P*_*a*_ is the probability that action *a* in state *s* will lead to state *s*′ and this is equal to the probability of some random state Pr, at time *t* + 1 equaling the new state *s*′. This extends Equation 15 to include time, and can be given as the in Equation 17 below:


(17) $$P_{a}\left(s,s^{\prime }\right)=\mathrm{Pr}\left(s_{t+1}=s^{\prime }|s_{t}=s,a_{t}=a\right),$$

A policy function π is then applied, which is a probabilistic mapping from state space to action space. State spaces and actions spaces can be infinite in real life, but need to be brought into a finite mapping space in order to be computable. This may explain why heuristics and quick processing systems of BIRR occur when making decisions, as finite spaces need to be mapped which are limited to how much time is available to process the information or stimuli. The goal of the Markov decision process is to find an optimal policy for the decision maker which maximizes the reward, given the change in state due to a given action (or decision), and the associated reward with such state change. Therefore, policy function π specifies an action for some state π(*s*), which the individual will decide when in state *s*. The policy decides the decisions which lead to states with the highest reward value (i.e., a move or decision selection).

Once the Markov decision process is combined to a given policy in this way, it then behaves as a Markov chain, as the sequential actions are chosen by the policy π(*s*), which is given by *Pr*⁡(*s*_*t* + 1_ = *s*′|*s*_*t*_ = *s*, *a*_*t*_ = *a*) of Equation. This can be denoted as follows:


$$\text{Policy}\pi \left(\text{s},\text{a}\right)=\mathrm{Pr}\left(s_{t+1}=s^{\prime }|s_{t}=s,a_{t}=a\right)$$

The total amount of rewards an agent will try to maximize can be given by:


(18) $$R_{t}=r_{t+1}+r_{t+2}+\ldots .+r_{T},$$

Where *r*_*t*+1_ is the reward received by the agent (individual) at time step *t*_*o*_ which performs some action (decision) *a*_*o*_ to move from one state to another. *r*_*t* + 2_ is same (reward received by the agent) but at time step *t*_1_. *r*_*T*_ is the reward the agent receives at the final time step *T*.

Further to this, a value *V* needs to be specified for state*s*, and this is given as *V*(*s*). *V*(*s*) contains the discounted sum of the expected rewards for the given paths from state *s*. For this, the MDP solution requires policy π is a mapping of each state, *s* ∈ *S*, and actions *a* ∈ *A*(*s*), to the probability π(*s*, *a*) of taking action *a* when in state *s* under a policy *V*_π_(*s*), gives the expected reinforcing return when starting at *s* and following a given policy π.

*V* then holds two arrays of data, one which contains the real values and the other which states the policy π which contains the actions of the optimal decision solution which maximizes *V*(*s*). Both the value and policy updates given each path and expected outcomes. *V*_π_(*s*) can be given as follows:


(19) $$V_{\pi }\left(s\right)=E_{\pi }\left\{\sum_{k=0}^{\infty }\gamma^{k}r_{t}+k+1|s_{t}=s\right\},$$

Where *E*_π_{}*s* denotes an expected value when following policy π, whilst *t* is any time step. *V*_π_ is called the *state-value function* for a given policy π. In a similar way, the *action-value function* for policy π defines the value of taking action (a decision) *a* in state *s*, under a given policy π, and can be denoted by *Q*_π_(*s*, *a*), as the expected reinforcing return when starting from *s*, and taking action *a*, whilst following a given policy π. This can be given as:


(20) $$\begin{array}{c} Q_{\pi }\left(s,a\right)=E_{\pi }\{R_{t}|s_{t}=s,a_{t}\\ =a\}=E{}_{\pi }\{\sum_{k=0}^{\infty }\gamma^{k}r_{t}+k+1|s_{t}=s,a_{t}=a\}, \end{array}$$

A Monte Carlo tree search (MCTS), which is similar to the MDP approach can be further applied for exploration and exploitation, of each state, i.e., to decide which actions (decisions) lead to the highest value in a given situation. This has the advantage over MDP as it is specifically utilized in situations where the outcomes in a process are not easily predicted due to the potential for random variables or other interventions. This MCTS specified that actions are made which has the highest value for the exploration and exploitation, which can be given as:


(21) $$\frac{w_{i}}{n_{i}}+\sqrt[{c}]{\frac{InN_{i}}{ni}},$$

This is typically applied in game situations with two or more players who can impact on the reward gained for the other (but this does not have to be the case). As maximizing a value can be considered a game, where strategies of actions improve states, then, *w*_*i*_ can be regarded as the number of wins (or optimal states), for the node considered in the *i*-th move. *n*_*i*_ is the number of simulations for the node considered in the *i*-th move, *N*_*i*_ is the total number of simulations, after the *i*-th move run by the parent node. *c* is the exploration parameter, theoretically equal to $\sqrt{2}$.

A game situation could be considered between two individuals who read a PH message, and are trying to optimize their reward. For example, in a sequence of steps, if one conforms to movement restriction and the second individual knows this, then that second individual may benefit from the reduced spread of COVID-19 whilst not adhering to movement restriction, therefore maximizing reward. This, therefore, may provide some further explanation for how biased decisions are made in complex and dynamic environments when there are two or more agents (players) involved.

Monte Carlo tree search includes four phases, selection, expansion, simulation, and backpropagation. For selection it starts at the root node, then moves down the tree by selecting the optimal “child” node until the last node is reached. At the expansion phase it then creates more child nodes according to the available actions at the current state node and selects the first of these new nodes. At simulation, a simulated rollout is run until a terminal state (game terminated) is found. The terminal state contains a value which is returned back in the backpropagation phase, and the nodes from root to new nodes are updated.

**FIGURE 8:**
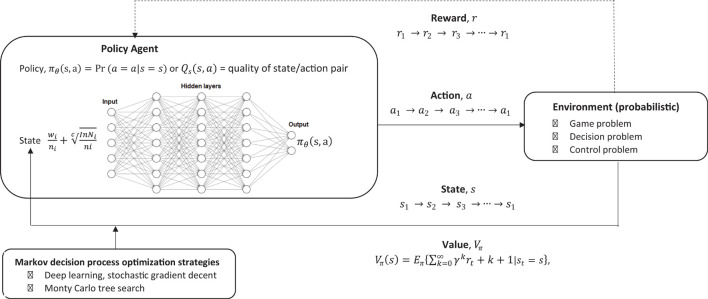
Overview of reinforcement learning machine learning framework, but with a deep neural network implemented as policy optimizer.

A deep neural network can also be applied as they are function approximators, and useful for large state space and actions spaces which include a high degree of uncertainty. In this way the neural network (typically convolution networks) can be used to approximate a value function or policy function through mapping state action pairs to Q values and expressed as *Q*_*s*_(*s*, *a*). It does this through using coefficients to approximate a function, through relating inputs and outputs or weights which iteratively adjust to minimize a gradient decent and error term through backpropagation (see Figure 8 for the updated policy which include MCTS with a deep neural network).

**FIGURE 9:**
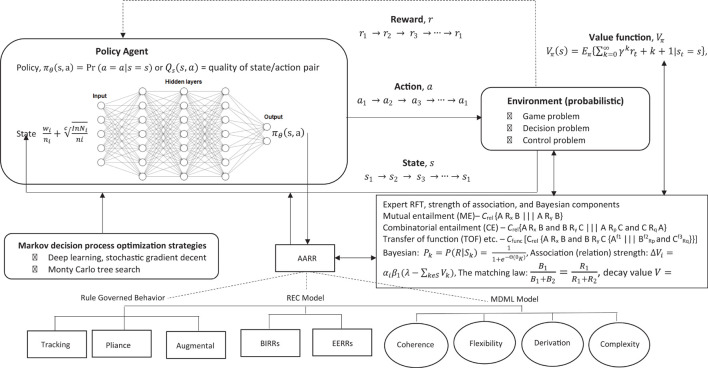
The REC RFT adapted version of the RL machine learning framework, with a deep neural network implemented as policy optimizer.

Finally, the RFT relational frames can be incorporated (see Figure 9) in a way which allows for starting state inputs to be fed into the policy π neural network, and receive action-state outputs which can be relationally networked, and fed back onto the policy network, constantly updating the policy in accordance to how stimuli is networked relationally. Some additional terms may need to be stated to ensure this approach accurately reflects updated versions of the RFT model, and can store longer term relational networks, but this at least may represent a solid starting point for further modeling work and implementation within a decision making context.

## Conclusion

The REC RFT approach within a mathematical framework of finding optimal decision policy through determining the value of particular action states, perhaps allows for the greatest degree of flexibility and predictive power when attempting to predict whether individuals will adhere to a PH message. As such, this review has outlined some potential uses of the BA RFT approach for modeling how effective PH messages maybe in promoting or nudging adherence toward the message. It has also identified ways in which this may go beyond the simpler heuristic accounts of Kahneman such as dual process theory and prospect theory, by identifying the context in which reinforcement learning occurs, through a relationally framed network.

This is encouraging, as PH agencies could perhaps benefit from utilizing this modeling approach more concretely in a way that would optimize the effectiveness of PH messaging to increase overall local and global adherence. If a PH agency were to adopt this approach, this would represent a move away from a complete human-centric processes as it currently stands i.e., intuition-based decision making, and a move toward more analysis-based decision making, for example, where a human is assisted by algorithmic modeling in the form of REC RFT in this case. This could then be further extended with use of visualization approaches, which map the predicted outcomes of the RFT network to facilitate better PH messaging, by increasing the ability of PH agencies to visualize the very complex predicted the public responses (whether global or local) to a particular PH message given in a particular context and frame.

By exploiting these approaches, this may ultimately help PH agencies identify and avoid potential bias which will likely lead to reduced adherence toward the PH message. Perhaps such an approach could then also be applied to other area such as economic decisions, political planning, and mental health wellbeing modeling of a society through visualizing the outputs of the RFT REC model. By creating models with high predictive and descriptive power such as REC RFT, this may ultimately save lives in the future, so it is of high importance that these approaches are further explored for the potential benefits they may provide in PH messaging application.

## Author Contributions

DE is solely responsible for all components of this manuscript.

## Conflict of Interest

The author declares that the research was conducted in the absence of any commercial or financial relationships that could be construed as a potential conflict of interest.

## Publisher’s Note

All claims expressed in this article are solely those of the authors and do not necessarily represent those of their affiliated organizations, or those of the publisher, the editors and the reviewers. Any product that may be evaluated in this article, or claim that may be made by its manufacturer, is not guaranteed or endorsed by the publisher.
